# The MALAT1 gene polymorphism and its relationship with the onset of congenital heart disease in Chinese

**DOI:** 10.1042/BSR20171381

**Published:** 2018-05-22

**Authors:** Qian Li, Wenying Zhu, Bei Zhang, Yiping Wu, Sen Yan, Yufeng Yuan, Haiyan Zhang, Jie Li, Kai Sun, Hua Wang, Tingting Yu

**Affiliations:** 1 Department of Pediatric Internal Medicine, The First People’s Hospital of Kunshan Affiliated to Jiangsu University, Kunshan 215300, China; 2 Department of Ultrasound Medicine, The Affiliated Hospital of Guizhou Medicial University, Guiyang 550002, China; 3 Department of Developmental Genetics, Nanjing Medical University, Nanjing 211166, China; 4 Department of Emergency Medicine, The First Affiliated Hospital with Nanjing Medical Universtiy, Nanjing 210009, China

**Keywords:** congenital heart disease, Lnc MALAT1, polymorphism, susceptibility

## Abstract

Many long non-coding RNAs (lncRNAs), including lncRNA metastasis-associated lung adenocarcinoma transcript 1 (MALAT1), are involved in various cardiac diseases. We evaluated the effects of tag single nucleotide polymorphisms (tag-SNPs) on *MALAT1* gene in a Chinese population of children with congenital heart disease (CHD). In the present study, 713 CHD patients and 730 gender- and age-matched children without CHD were genotyped for MALAT1 tag-SNPs rs11227209, rs619586, and rs3200401. Further investigation of SNP’s function was performed by luciferase assay. Statistical analyses, including uni- and multivariate logistic regression were performed to quantitate the association between these tag SNPs and CHD. We discovered that MALAT1 rs619586 GG allele was significantly associated with lower risk of CHD (odds ratio (OR) = 0.77, 95% confidence interval (CI) = 0.59–0.92, *P*=0.014) in additive model. Functional investigation indicated that G allele of rs619586 could trigger higher expression of MALAT1. We demonstrated that the functional MALAT1 polymorphism rs619586 A>G was significantly associated with CHD susceptibility in Chinese population, potentially via regulating MALAT1 expression.

## Introduction

Congenital heart disease (CHD) is a common birth defect in children. Prevalence of birth with CHD is approximately 8 per 1000, however there is a large variation in different areas in the world [1]. Estimated CHD prevalence is 4 per 1000 in adults in comparison [2]. Clinically, atrial septal defect (ASD), ventricular septal defect (VSD), patent ductus arteriosus (PDA), pulmonary stenosis (PS), tetralogy of Fallot (TOF), transposition of the great arteries (TGA), and aorta stenosis (AoS) are the common subtypes of CHD [3]. CHD is the leading cause of childhood deaths with approximately 1% annual mortality rate, and victims are usually under the age of 5 [2].

CHD results from complex consequences of structural abnormality of heart or intrathoracic great vessels [4]. As Corno et al. [5] reported, hypoxia was considered as significant trigger to CHD in the early stage of birth; hypoxia-inducible factors (HIFs) were imperative transcription factors and/or regulator involved in CHD [6]. Based on this molecular mechanism, pulmonary arterial hypertension (PAH) is a severe disease associated with CHD, and PAH-CHD is commonly associated with poor prognosis [7,8].

Modern molecular biology has identified non-coding RNAs (ncRNAs) as important factors in CHD onset [9–11]. ncRNAs could be further divided into two categories according to their lengths: small ncRNAs with length less than 200 nt, including miRNAs, and longer ncRNAs with length over 200 nt, including long ncRNAs (lncRNAs) [12,13]. While miRNAs and their molecular mechanisms have been well studied in heart diseases [14–16], little has been explored or understood on lncRNAs’ role in CHD pathogenetics.

Metastasis-associated lung adenocarcinoma transcript 1 (MALAT1) is a special lncRNA that influences epigenetic regulation and splicing [17,18]. In heart diseases, MALAT1 regulates cardiomyocyte and endothelial cell proliferation, both are crucial for CHD onset [19–21].

Single nucleotide polymorphisms (SNPs) in lncRNA were reported to alter the function of their corresponding lncRNA by directly regulating lncRNA expression [22–24]. It has been reported that functional polymorphism of MALAT1 is associated with PAH susceptibility in Chinese population [25]. Considering the imperative function of MALAT1 in heart disease and the general role of SNPs, we performed a case–control study in Chinese children to identify and quantitate the association between MALAT1 polymorphism and CHD.

## Materials and methods

### Study population

Two hundred and forty seven CHD patients and 270 controls without CHD were enrolled in the present study, all of whom were 1–60 months children and genetically unrelated Han Chinese. All participants were recruited from the First People’s Hospital of Kunshan affiliated to Jiangsu University, between January 2015 and December 2016. All cases were diagnosed by ultrasound. Cases with structural malformations in other organs, or known chromosomal, abnormalities, or other heart-related disease were excluded. Control group were age- and gender-matched healthy children without congenital cardiac or other diseases, who were in the same geographic area and born in the same period as controls.

The present study was approved by the Ethics Committee of the First People’s Hospital of Kunshan affiliated to Jiangsu University (approval number 201705), in accordance with the principles of the Helsinki Declaration. Each statutory guardian of the participant signed a written informed consent before donating 5 ml venous blood of their children for further investigation and analysis. Participants’ demographic and clinical characteristics were also recorded.

### SNP selection

MALAT1 SNPs were selected based on the analysis of the gene in Haploview 4.2 software. The following criteria were adopted to select the potential SNPs. First, the candidate SNP should locate within the *MALAT1* gene region. Second, the minor allele frequency (MAF) of the selected SNP should be above 0.05 in Chinese Han Beijing (CHB) population. Third, linkage disequilibrium value *r^2^* should be less than 0.8 for the candidate SNP. In addition, MALAT1 SNPs investigated in our previous research were also included in the present study.

### DNA isolation

Genomic DNA of each sample (5 ml peripheral venous blood) was extracted using QIAamp DNA blood mini kit (Qiagen, Dusseldorf, Germany) following manufacturer’s protocol. We assessed A260/A280 ratio of the purified DNA samples to ensure purity. DNA samples were then stored at −80°C for further genotyping.

### Genotyping

Genotyping of SNPs was performed with Taqman probes on ABI 7900HT real-time PCR system (Applied Biosystem Inc, Foster City, CA, U.S.A.). Three samples were chosen as genotyping controls and added into each 96-well plate. Ten percent of these DNA samples were selected randomly for further validation. Two researchers performed genotyping independently to cross-validate the results. The imputation of SNPs was then performed with IMPUTE v2.3.2 software, with 1000 Genomes Phase 3 as reference data, according to The Cancer Genome Atlas (TCGA) SNP array genotype program [26].

### Statistical analysis

χ^2^ goodness-of-fit test was adopted to derive the Hardy–Weinberg equilibrium (HWE). In the case–control study, Student *t* test and/or χ^2^ test were used to demonstrate how demographic and clinical characteristics and frequency of genotypes differed between case and control groups. Unconditional univariate and multivariate logistic regressions were applied to evaluate the effects of SNPs and to quantitate the association between the SNPs and CHD. Adjusted odds ratios (ORs) and with 95% confidence intervals (CIs) were calculated. The imputation of SNPs was finally performed with the assistant of IMPUTE v2.3.2 software, with the 1000 Genomes Phase 3 as reference data, according to TCGA SNP array genotype program [26]

## Results

### Characteristics of study participants

Demographic and clinical characteristics of the 713 CHD patients and 730 disease-free controls in the present study are provided in Table 1. There were no statistically significant differences in age and gender between CHD patient and control groups (*P*=0.819 and *P*=0.651, respectively), indicating these two factors were well-matched in the present study. In parents’ characteristics, fathers’ ages showed no significant difference between CHD case and control group (28.6 ± 4.9 compared with 29.1 ± 5.1, case compared with control, *P*=0.058), however there was some difference in the age of mothers (27.9 ± 5.4 compared with 28.5 ± 5.2, *P*=0.032). There was no significant difference in the history of parents with chronic diseases between CHD case and control groups (*P*=0.724). More mothers reported they had smoked (*P*=0.042) or drunk (*P*=0.023) during their pregnancy in CHD group than control group.

**Table 1:** Characteristic and selected variables in CHD cases and controls

Characteristics	Case	Control	*P*-value
Children	*n*=713	*n*=730	
Gender			0.819
Male	361	374	
Female	352	356	
Age (months)	41.9 ± 26.9	40.7 ± 20.8	0.651
Parents (age, years)			
Father	28.6 ± 4.9	29.1 ± 5.1	0.058
Mother	27.9 ± 5.4	28.5 ± 5.2	**0.032**
History of chronic illnesses	75	81	0.724
Mother smoking	71	51	**0.042**
Mother drinking	83	59	**0.023**
CHD types (*n*, %)			
VSD	496		
ASD	103		
TOF	51		
PDA	24		
PAH	5		
DORV	15		
PS	8		
AS	6		

Abbreviations: AS: aortic stenosis; ASD: atrial septal defect; CHD: congenital heart disease; DORV: double outlet right ventricle; PAH: pulmonary arterial hypertension; PDA: patent ductus arteriosus; PS: pulmonary stenosis; TOF: tetralogy of Fallot; VSD: ventricular septal defect;

Bold text indicates *P* < 0.05

### Association between MALAT1 SNPs and the risk of CHD

Three candidate SNPs in MALAT1 (rs11227209, rs619586, and rs3200401) were selected according to the aforementioned criteria. In control group, genotypes were consistent with the expected values from HWE for all three SNPs (*P*=0.531 for rs11227209, *P*=0.107 for rs619586, and *P*=0.377 for rs3200401). After adjustment for age and gender, detailed information of MALAT1 polymorphisms were shown in Table 2. There was a significant association between rs619586 and the risk of CHD. In dominant model, AG/GG genotypes were associated with significantly lower risk of CHD compared with AA genotype (adjusted OR = 0.76, 95% CI = 0.59–0.98). Neonates with GG genotype of rs619586 also had substantially lower risk of CHD (OR = 0.09, 95% CI = 0.02–0.78). And in additive model of rs619586, the protective effect of this SNP was also significant (adjusted *P*=0.014, OR = 0.77, 95% CI = 0.92). There was no statistically significant association between SNPs rs11227209/rs3200401 and CHD risk (adjust *P*=0.573 and 0.423 in additive model, respectively).

**Table 2:** Genotypes of MALAT1 polymorphisms in CHD cases and controls

	Genotypes (AA/Aa/aa)		Adjusted OR (95% CI)^1^					*P*-value in additive model
SNPs	Cases (*n*=713)	Controls (*n*=730)	Co-dominant model		Dominant model	Recessive model	Additive model	
			Aa compared with AA	aa compared with AA	Aa + aa compared with AA	aa compared with AA + Aa	AA compared with Aa compared with aa	
rs11227209 C>G	656/57/0	660/65/4	0.89 (0.67–1.21)	N/A	0.81 (0.63–1.16)	N/A	0.72 (0.58–1.11)	0.573
rs619586 A>G	639/72/2	624/100/6	0.83 (0.61–1.03)	0.15 (0.05–0.79)	0.76 (0.59–0.98)	0.09 (0.02–0.78)	0.77 (0.59–0.92)	**0.014**
rs3200401 C>T	508/190/14	519/192/18	1.02 (0.86–1.23)	0.78 (0.45–1.32)	0.98 (0.81–1.21)	0.79 (0.44–1.34)	0.92 (0.81–1.04)	0.423

1 Adjusted for age and sex in logistic regression model.

Bold text indicates *P* < 0.05.

### Stratified analysis of rs619586 polymorphism in CHD clinical characteristics

Stratified analysis to investigate rs619586 and CHD clinical characteristics was performed and the detailed results were listed in Table 3. AG/GG genotypes of rs619586 were associated with significantly lower risk of CHD subtype VSD against AA genotype (adjusted *P*=0.011, OR = 0.72, 95% CI = 0.54–0.92). However, AG/GG genotypes did not differ significantly from AA genotype on subtype ASD and all other CHD subtypes (*P*=0.314 and *P*=0.065, respectively).

**Table 3.** Stratified analysis of MALAT1 rs619586 polymorphism

Types	AA		AG/GG		Adjusted OR (95%CI)^1^	*P*^1^
	Case	Control	Case	Control		
VSD	447	624	49	106	0.72 (0.54–0.92)	**0.011**
ASD	92	624	11	106	0.83 (0.62–1.12)	0.314
Others	100	624	9	106	0.77 (0.57–1.01)	0.065

1 Adjust for age and gender in logistic regression model.

Bold text indicates *P* < 0.05.

### Impact of MALAT1 polymorphisms on MALAT1 expression

**Figure 1:**
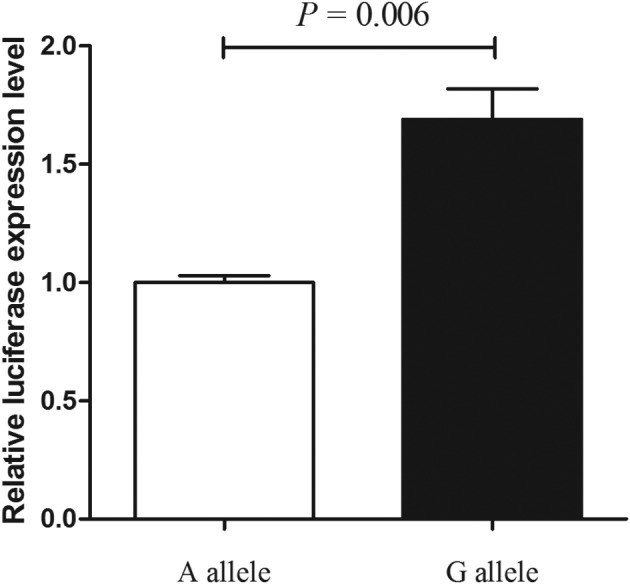
The luciferase assay of MALAT1 rs619586 A/G polymorphisms

We explored the molecular mechanism of rs619586 (transcriptional function) modulating MALAT1 expression. Results of luciferase assay demonstrated that G allele of rs619586 dramatically decreased the luciferase activity in primary cardiac myocyte (Figure 1), suggesting that rs619586 G allele lowered the expression of MALAT1 in myocardial cells, and consequently decreased the risk of CHD.

## Discussion

Our study demonstrated that MALAT1 was associated with CHD onset; higher expression of MALAT1 in plasma was found in CHD patients. Polymorphisms in *MALAT1* gene was genotyped and investigated for their associations with CHD occurrence in a Chinese population of children. We identified that rs619586, an MALAT1 tag-SNP, was significantly protective against CHD. Further investigation showed that G allele of rs619586 decreased MALAT1 expression *in vivo*. These results suggested that MALAT1 polymorphism could be a pivotal genetic risk factor for CHD.

In hypoxic conditions, MALAT1 was found to be an overencoded highly conserved lncRNA [27]. MALAT1 was a protective mediator and reduced apoptosis of cardiomyocyte [28]. In our study, MALAT1 SNP rs619586 was associated with significantly lower CHD risk, and this discovery coincided with the conclusion from prior investigation of PAH patients in a Chinese population [25]. Our following *in vivo* study also revealed higher luciferase activity with MALAT1 rs619586 G allele. These results indicated that rs619586 G allele was the key to decrease the risk of CHD by up-regulating MALAT1 expression.

Novel perspective has been established for MALAT1 in PAH pathogenesis. MALAT1 with rs619586 G allele could get more affinity with *miR-214* [25] as competing endogenous RNA (ceRNA), and abrogated the regulative relationship between *miR-214* and XBP1. However, to our knowledge, relationship between expression of both MALAT1 and *miR-214* in PAH and heart disease (such as the acute myocardial infarction) was not comprehensively studied [28,29]. We also evaluated MALAT1 and *miR-214* expression in the CHD patients’ plasma (Supplementary Figure S1), although it was not the ideal measurement of ncRNA in CHD patients. According to ceRNA mechanism, the overexpressed MALAT1 should recruit more *miR-214* and lead to a lower *miR-214* level. However, overexpression of MALAT1 and *miR-214* were both identified in CHD patients carrying different genotypes of rs619586. It was contrary to the conclusion that MALAT1 rs619586 G allele was associated with higher risk of PAH via directly regulating the *miR-214* [25]. Since the protective function of MALAT1 was established, we believed that the function of rs619586 was not involved in regulating the relation of ceRNA between MALAT1 and *miR-214*, at least in CHD.

Several limitations are needed to be pointed out in the present study. First, because the participants were all enrolled from local hospital, selection and information bias were inevitable and we used stratified analysis as a remedy. Second, the relative small sample size of our study might affect the statistical power, especially for the stratification analysis. In addition, due to positive interventions and increased socio-economic development, approximately 90% of children with CHD could survive to their adulthood. However, corresponding follow-up information, which could be valuable for CHD investigation, has not been updated.

## Conclusion

In conclusion, our study identified the significantly protective effect of MALAT1 rs619586 A>G polymorphism on CHD in a Chinese population. We also established the molecular mechanism between SNP rs619586 and *MALAT1* gene expression. We suggest that MALAT1 rs619586 polymorphism could be a novel and effective biomarker for CHD.

## Supporting information

## Abbreviations

AS: atrial stenosis
ASD: atrial septal defect
ceRNA: competing endogenous RNA
CHD: congenital heart disease
CI: confidence interval
DORV: double outlet right ventricle
HWE: Hardy–Weinberg equilibrium
lncRNA: long non-coding RNA
MALAT1: metastasis-associated lung adenocarcinoma transcript 1
ncRNA: non-coding RNA
OR: odds ratio
PAH: pulmonary arterial hypertension
SNP: single nucleotide polymorphism
TCGA: The Cancer Genome Atlas
VSD: ventricular septal defect
