# Supplementary figures and images for: The MALAT1 gene polymorphism and its relationship with the onset of congenital heart disease in Chinese
Source: Biosci Rep. 2018 May 22;38(3):BSR20171381. doi: 10.1042/BSR20171381 (PMC6048208; doi:10.1042/BSR20171381)

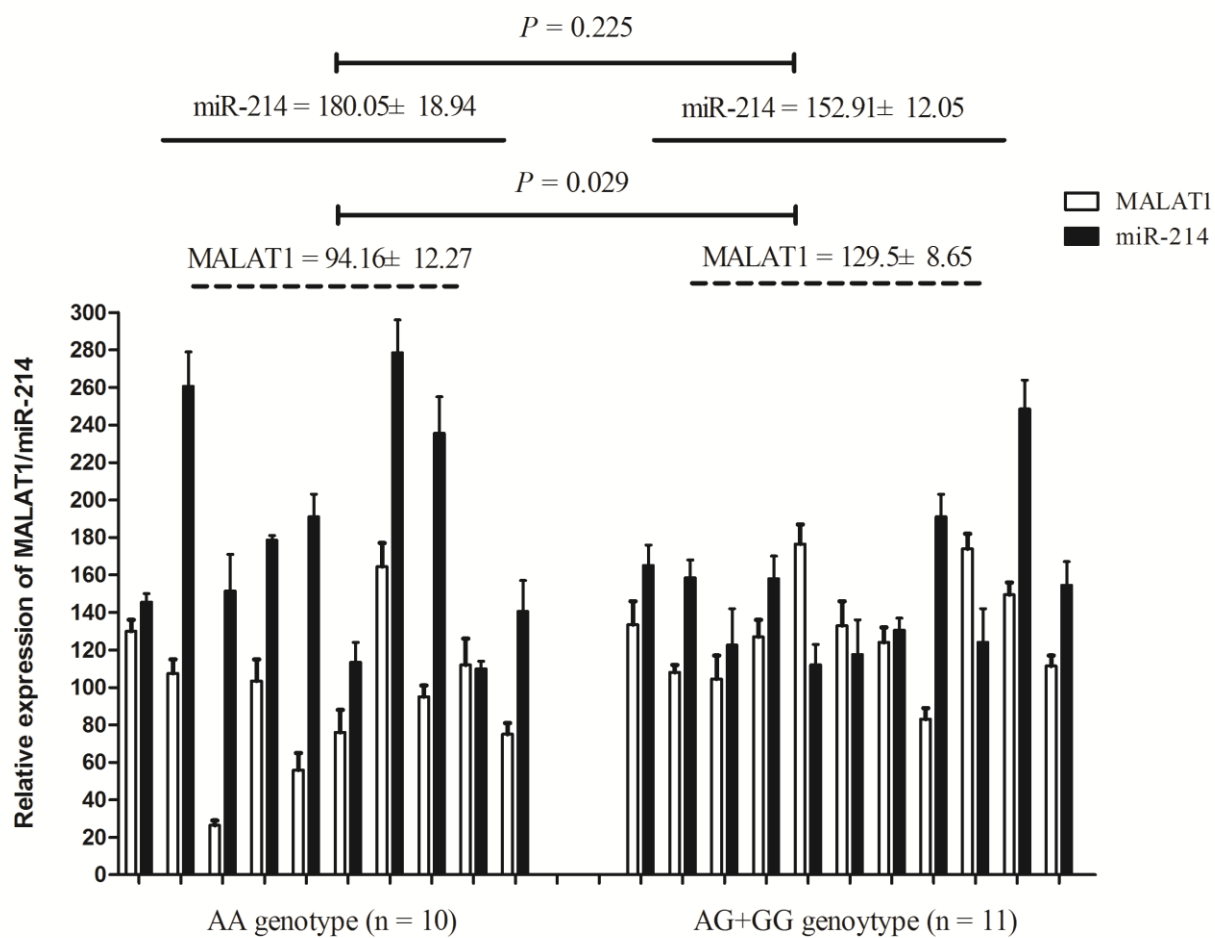

Supplement: Supplementary file 1 [file bsr20171381_Supp1.pdf]
